# Simultaneous robot‐assisted laparoscopic radical prostatectomy and inguinal hernia repair using a polypropylene mesh: Report of two cases

**DOI:** 10.1002/iju5.12615

**Published:** 2023-07-27

**Authors:** Shugo Yajima, Yasukazu Nakanishi, Kohei Hirose, Sao Katsumura, Madoka Kataoka, Hitoshi Masuda

**Affiliations:** ^1^ National Cancer Center Hospital East Chiba Japan

**Keywords:** inguinal hernia, polypropylene, prostate cancer, radical prostatectomy, robotic surgery

## Abstract

**Introduction:**

We report two cases where robotic‐assisted laparoscopic radical prostatectomy and inguinal hernia repair were performed simultaneously.

**Case presentation:**

In case one, hernia repair was performed by implantation of 3D Max™ mesh and closure of the peritoneum. Total console time was 156 min, of which hernia repair took 21 min. In case two, hernia repair was performed using Ventralight™ ST mesh. Total console time was 181 min, of which hernia repair took 23 min. Pelvic lymph node dissection was performed in case two but not in case one. Both patients were discharged 7 days postoperatively without severe complications or mesh infection.

**Conclusion:**

It may be possible to perform robotic‐assisted laparoscopic radical prostatectomy and inguinal hernia repair simultaneously.

**Abbreviations & Acronyms**
CT: computed tomography
RARP: robot‐assisted laparoscopic radical prostatectomy
R‐TAPP: robot‐assisted transabdominal preperitoneal inguinal hernia repair

**Keynote message**

- We report a case in which robot‐assisted laparoscopic radical prostatectomy and inguinal hernia repair using a polypropylene mesh was performed simultaneously.
- The extensive incision of the peritoneum during pelvic lymph node dissection made it impossible to close the peritoneal flap, but hernia repair was successfully done by sewing an artificial polypropylene mesh over the hernia area in a circumferential fashion.
- Console times for hernia repair were 23 minutes, suggesting that simultaneous robot‐assisted laparoscopic radical prostatectomy and hernia repair may result in considerable time savings, and no severe complications or mesh infection occurred in the case reported here.

## Introduction

RARP is a widely used treatment for prostate cancer that is safe and effective. However, patients who undergo RARP have a 7%–21% risk of developing an inguinal hernia after surgery.^1^ Moreover, prostate cancer and inguinal hernia may coexist because of the bimodal distribution of inguinal hernia incidence, which peaks around 5 years after surgery for people over 70 years of age and is more common in males.^2^ Several studies have reported on RARP combined with R‐TAPP, and a growing body of evidence supports the safety and feasibility of this combination.^3^

Similarly, we report two cases in which RARP and R‐TAPP were performed simultaneously. The unique aspect of this report is that in one case, extensive incision of the peritoneum during pelvic lymph node dissection made it impossible to close the peritoneal flap, but hernia repair was successfully completed by sewing an artificial polypropylene mesh over the hernia area in a circumferential fashion.

## Case

Herein, we report two cases of simultaneous RARP and R‐TAPP performed at our institution. The operations were performed with a 6‐port transperitoneal technique by one highly skilled surgeon using a Da Vinci Xi surgical system (Intuitive Surgical Inc., Sunnyvale, CA, USA). We performed preoperative urine cultures in both cases, which were negative.

### Case 1

**Fig. 1:**
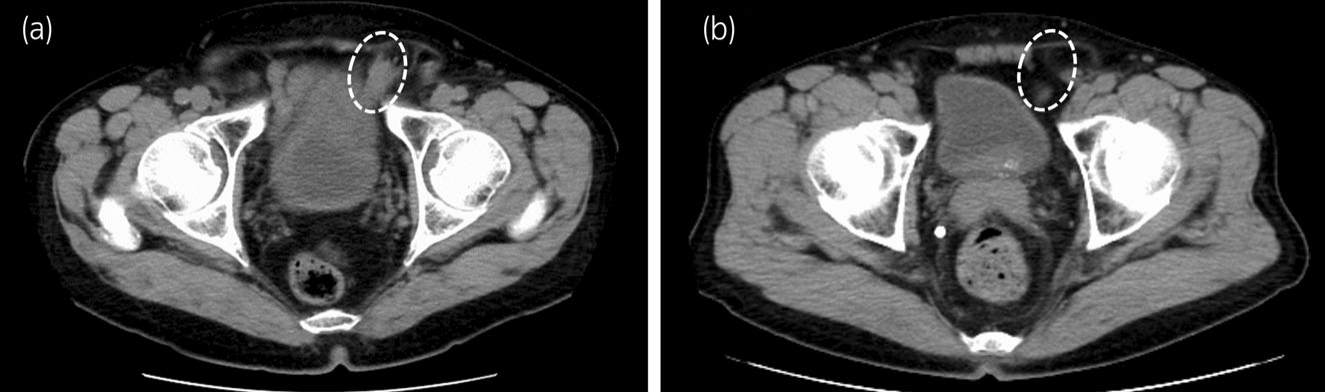
Preoperative CT confirmed left direct inguinal hernias (broken white circles) in both Case 1 (a) and Case 2 (b).

**Fig. 2:**
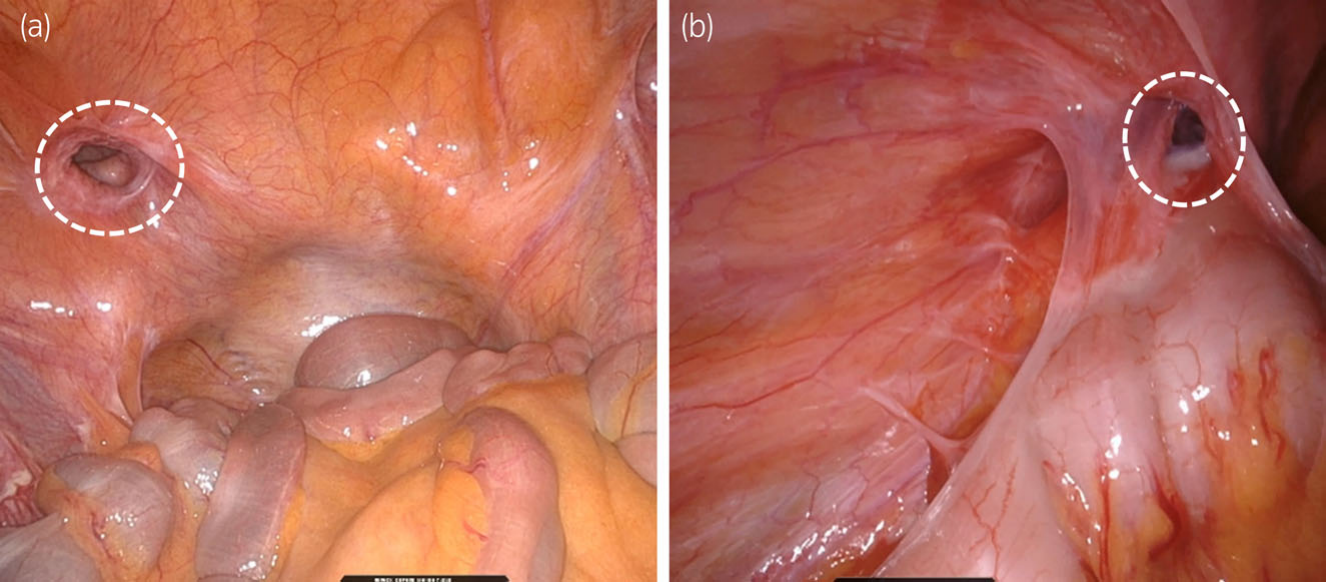
Intraperitoneal views of the direct inguinal hernias in both Case 1 (a) and Case 2 (b). Broken white circles indicate the hernial orifices.

**Fig. 3:**
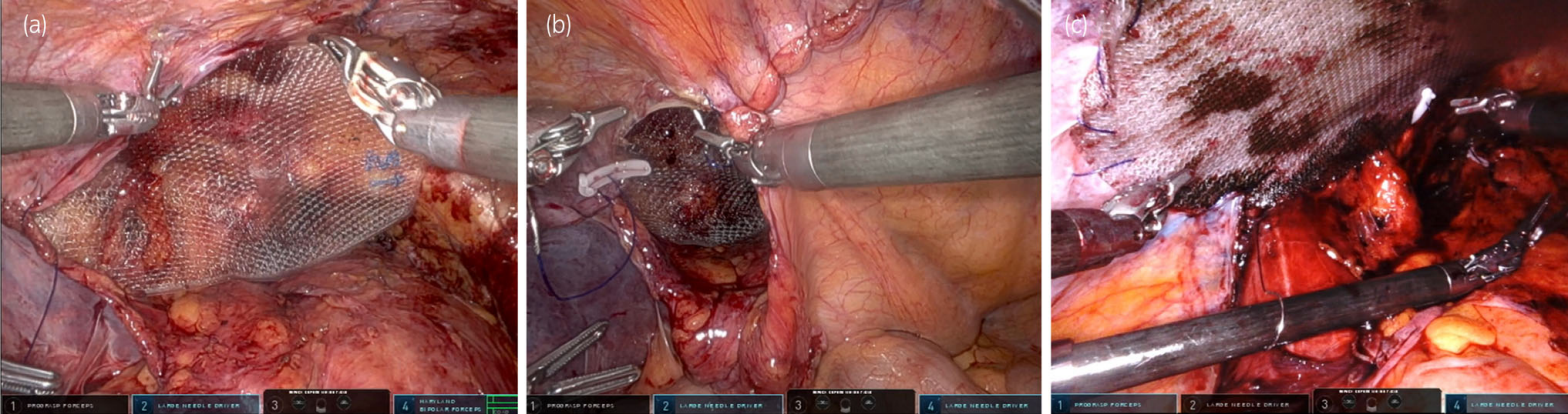
In Case 1, a 3D Max™ mesh was fixed over the hernial orifice using two stitches (a), and the peritoneal flap was repaired with continuous sutures (b). In Case 2, the peritoneum could not be repaired, and a Ventralight™ ST mesh was placed over the hernial orifice and sutured circumferentially (c).

Case one was a 69‐year‐old man diagnosed with prostate cancer cT1cN0M0, Gleason score 4 + 4, and a left‐sided symptomatic inguinal hernia (Fig. 1a). He had a history of gallbladder adenomyomatosis and had undergone a laparoscopic cholecystectomy. He underwent simultaneous RARP and R‐TAPP. The intraperitoneal view of the direct inguinal hernia is shown in Figure 2a. First, a horizontal incision was made in the peritoneum about 6 cm above the hernia defect, and the line of dissection was carried laterally. On the way to reach the Retzius' cavity, the hernia sac was isolated from the peritoneum and dissected. Subsequently, prostatectomy and vesicourethral anastomosis were performed. Then, a 3D Max™ mesh (Bard, Inc, New Providence, NJ) was placed over the hernial orifice, two stitches were used for fixation (Fig. 3a), and the peritoneal flap was repaired with continuous sutures (Fig. 3b). Total console time was 156 min, of which hernia repair took 21 min. The estimated blood loss was 313 mL. The patient was discharged 7 days postoperatively without severe complications (Clavien‐Dindo grade 3 or more).

### Case 2

Case two was a 73‐year‐old man diagnosed with prostate cancer cT3bN0M0, Gleason scores 5 + 4, and a left‐sided symptomatic inguinal hernia (Fig. 1b). He had a medical history of hypertension. He underwent R‐TAPP concurrently with RARP and pelvic lymph node dissection. The intraperitoneal view of the direct inguinal hernia is shown in Figure 2b. As in case one, a short horizontal incision was initially made into the extraperitoneal space, and the hernia sac was isolated from the peritoneum and dissected. Then, the incision was continued to the lymph node dissection area. Following that, we performed pelvic lymph node dissection, prostatectomy, and vesicourethral anastomosis. We subsequently performed a hernia repair. The peritoneum was not repaired because of the extensive peritoneal incision. Instead, a Ventralight™ ST mesh (Bard, Inc, New Providence, NJ, USA) was applied over the hernial orifice using continuous sutures (barbed) in a full circumferential fashion. The ventral side was sutured to the peritoneum, and the dorsal side to the pectineal ligament (Fig. 3c). Total console time was 181 min, of which hernia repair took 23 min. The estimated blood loss was 18 mL. The patient was discharged 7 days postoperatively without severe complications (Clavien‐Dindo grade 3 or more).

Over a 3‐month period, both cases progressed well with no hernia recurrence or mesh infection.

## Discussion

We described two cases of simultaneous R‐TAPP and RARP. In general transabdominal pre‐peritoneal hernia repair, the peritoneum is incised, a mesh is placed, and the peritoneum is closed with barbed sutures.^4^ In Case 2, we repaired a hernia successfully using a Ventralight™ ST mesh, even though it was difficult to close the peritoneum.

Considering that the mean operative time and the mean console time were 114.8 and 87 min, respectively, in a case series where R‐TAPP was performed after RARP,^5^ it is safe to assume that simultaneously performing RARP and hernia repair resulted in considerable time savings. This may be because the hernia repair was done in an optimal situation, without any changes in the abdominal wall or inguinal structures that might occur after prostatectomy.

The R‐TAPP has less postoperative pain than other methods, such as open or laparoscopic,^4^ and may become more popular in the future. In our present study, the extra time needed for R‐TAPP was 21 and 23 min, which is not very different from the time of 26 min in a prior meta‐analysis.^6^ Besides, our findings corroborate the meta‐analysis that reported no drawbacks of concurrent R‐TAPP in terms of a higher risk of bleeding or postoperative complications.^6^ Our report provides one piece of evidence that simultaneous RARP and R‐TAPP can be performed safely. However, we acknowledge that more controlled prospective studies are needed to verify the safety and efficacy of this procedure. Moreover, our follow‐up period was short (about 3 months), and a previous case series also had a short follow‐up period (about 12 months).^1^ Therefore, long‐term follow‐up data on hernia recurrence is essential. It should also be noted that while the cost of the mesh used during surgery was reimbursed in this report, the fee for the inguinal hernia repair cannot be billed to insurance when RARP and inguinal hernia repair are done at the same time, under the Japanese insurance system.

## Author contributions

Shugo Yajima: Conceptualization; writing – original draft. Yasukazu Nakanishi: Supervision; writing – review and editing. Kouhei Hirose: Writing – review and editing. Sao Katsumura: Writing – review and editing. Madoka Kataoka: Writing – review and editing. Hitoshi Masuda: Supervision; writing – review and editing.

## Conflicts of interest

The authors declare no conflict of interest.

## Approval of the research protocol by an Institutional Reviewer Board

Not Applicable.

## Informed consent

Written informed consent for the release of this case report and accompanying images has been obtained from the patient.

## Registry and the Registration No. of the study/trial

Not Applicable.
